# A recommended early goal-directed management guideline for the prevention of hypothermia-related transfusion, morbidity, and mortality in severely injured trauma patients

**DOI:** 10.1186/s13054-016-1271-z

**Published:** 2016-04-20

**Authors:** Ryan Perlman, Jeannie Callum, Claude Laflamme, Homer Tien, Barto Nascimento, Andrew Beckett, Asim Alam

**Affiliations:** Department of Anesthesia, Rm M3-200, Sunnybrook Health Sciences Centre, University of Toronto, 2075 Bayview Avenue, Toronto, ON M4N 3M5 Canada; Trauma, Emergency & Critical Care Research Program, Sunnybrook Health Sciences Centre, University of Toronto, 2075 Bayview Avenue, Toronto, ON M4N 3M5 Canada; Department of Laboratory Medicine & Pathobiology, University of Toronto, 2075 Bayview Avenue, Toronto, ON M4N 3M5 Canada; Department of Clinical Pathology, Sunnybrook Health Sciences Centre, University of Toronto, 2075 Bayview Avenue, Toronto, ON M4N 3M5 Canada; Department of Surgery, Sunnybrook Health Sciences Centre, University of Toronto, 2075 Bayview Avenue, Toronto, ON M4N 3M5 Canada; Ornge—Ontario Air Ambulance, 5310 Explorer Drive, Mississauga, ON L4W 5H8 Canada; Department of Surgery, McGill University, Montreal General Hospital, 1650 Avenue Cedar, Montréal, QC H3G 1A4 Canada

## Abstract

Hypothermia is present in up to two-thirds of patients with severe injury, although it is often disregarded during the initial resuscitation. Studies have revealed that hypothermia is associated with mortality in a large percentage of trauma cases when the patient’s temperature is below 32 °C. Risk factors include the severity of injury, wet clothing, low transport unit temperature, use of anesthesia, and prolonged surgery. Fortunately, associated coagulation disorders have been shown to completely resolve with aggressive warming. Selected passive and active warming techniques can be applied in damage control resuscitation. While treatment guidelines exist for acidosis and bleeding, there is no evidence-based approach to managing hypothermia in trauma patients. We synthesized a goal-directed algorithm for warming the severely injured patient that can be directly incorporated into current Advanced Trauma Life Support guidelines. This involves the early use of warming blankets and removal of wet clothing in the prehospital phase followed by aggressive rewarming on arrival at the hospital if the patient’s injuries require damage control therapy. Future research in hypothermia management should concentrate on applying this treatment algorithm and should evaluate its influence on patient outcomes. This treatment strategy may help to reduce blood loss and improve morbidity and mortality in this population of patients.

## Background

Major or severe trauma is the primary cause of death in up to 10 % of all deaths worldwide. It is defined as having an Injury Severity Score (ISS) of 15 or greater [1]. Inadvertent injuries are the sixth leading cause of death and the fifth leading cause of severe disability internationally [1]. Hypothermia has been shown to exacerbate morbidity and mortality in severely injured trauma patients. Dr Benjamin Rush, the surgeon-general of military hospitals, first described this phenomenon during the American Revolutionary War. Within that time period, he eventually prohibited wet clothing for injured soldiers in order to avoid more serious complications [2]. Cold-related injuries were further described in World War I, World War II, and the Vietnam War as causing significant morbidity and mortality and effectively altering the outcome of several strategic encounters [3]. The lessons learned from these conflicts led to a change in over 30 clinical practice guidelines in combat conflicts, including the Iraq War. This protocol incorporated damage control resuscitation with performance improvement initiatives and led to a decrease in the rate of hypothermia from 7 % to 1 % [4].

Unfortunately, the adoption of civilian hypothermia prevention has been slower, necessitating the use of military data to augment our understanding. In 1987, Luna et al. [5] evaluated 94 trauma patients at a regional trauma center and discovered that hypothermia occurred in two-thirds of the cohort. Core temperature has been subsequently found to be inversely related to mortality regardless of the incidence of shock, resuscitation fluid, or ISS [6]. Gentilello et al. [7] confirmed that hypothermia was associated with serious morbidity and mortality in trauma (7 % vs. 43 % mortality) in a randomized trial. More importantly, however, reducing the period of hypothermia increased the probability of successful resuscitation.

Heat loss typically occurs at a rate of 60–75 kcal/h by four different methods: radiation, conduction, evaporation, and convection, as detailed in Table 1 [8]. Amongst trauma patients, heat loss is increased to 400 kcal/h and even mild hypothermia can result in significant morbidity [8]. A separate classification system has therefore been defined for this patient population (Table 2): mild hypothermia (34–36 °C), moderate hypothermia (32–34 °C), and severe hypothermia (<32 °C), where normothermia is 37 ± 0.5 °C [7, 8]. The efficacy of these temperature guidelines has not been formally investigated, but they have been adopted universally because of the negative physiological impact of hypothermia below 32 °C. Danzl et al. [9] aptly described this effect in their multicenter survey where severe hypothermia was associated with 84.9 % of casualties.

**Table 1:** Mechanisms of heat transfer in trauma

Mechanism	Rate (kcal/h)	Description
Radiation	10–50	Transfer of heat energy via electromagnetic waves down a concentration gradient without direct contact according to Boltzmann’s equation:^a^ *Q* = *K*(*T1* – *T2*)
		Methods to reduce losses include:
		• warming blankets
		• increasing environmental temperature
		• radiant heaters
		• avoid unnecessary anesthesia
Conduction	16–30	Transfer of energy between two solid objects in contact according to Fourier’s equation:^b^ *Q* = *KA* d*t*/d*x*
		Methods to reduce losses:
		• removal of wet clothing
		• avoid prolonged contact with cold surfaces
Convection	10–20	Transfer of heat energy during the mass movement of gas or liquid.
Evaporation	12–16	Heat energy transferred during change of phase (water to gas): 58 kcal/g water evaporated from skin, respiratory tract, and viscera
		Methods to reduce losses for convection and evaporation:
		• avoid prolonged surgery with an open abdomen
		• warming blankets

^a^ *Q* = rate of radiant heat transfer, *K* = a constant, *T1* = temperature of the first object, *T2* = temperature of the second object

^b^ *Q* = rate of heat transfer by conduction, *K* = thermal conductivity, *A* = area in contact, d*t*/d*x* = thermal gradient

Adapted with permission from [71]

**Table 2:** Classification of hypothermia

Classification	Conventional	Trauma patient
Mild hypothermia	35–32 °C (95.0–89.6 °F)	36–34 °C (96.8–93.2 °F)
Moderate hypothermia	32–28 °C (89.6–82.4 °F)	34–32 °C (93.2–89.6 °F)
Severe hypothermia	28–20 °C (82.4–68.0 °F)	32 °C (89.6 °F)
Profound hypothermia	20–14 °C (68.0–57.2 °F)	

Taken with permission from [72]

Damage control resuscitation focuses on the treatment of acidosis, coagulopathy, and hypothermia, also known as the “lethal triad”, in lieu of early definitive management [10]. While treatment guidelines already exist for acidosis and bleeding, there is no evidence-based systematic approach for managing hypothermia in trauma patients. This review will attempt to consolidate the evidence surrounding hypothermia management in trauma patients and propose a goal-directed treatment algorithm to prevent further morbidity and mortality.

### Evidence for temperature monitoring sites in trauma

Routine monitoring of temperature in severely injured patients is considered basic, but most techniques have not been validated in trauma patients. The most reliable core monitoring sites include the pulmonary artery, distal esophagus, nasopharynx, and tympanic membrane.

Swan and Ganz introduced the pulmonary artery catheter (PAC) in 1971 for measuring cardiac pressures. The PAC is the most accurate monitor and correlates to within 0.1 °C of core temperature [11, 12]. However, its routine use is often precluded in trauma patients because it is technically cumbersome to insert and is associated with its own complications, including arrhythmias, perforation, and pericardial tamponade [13]. The PAC has not been shown to improve clinical outcomes and has fallen out of favor for routine use in most noncardiac intensive care situations [14].

The oral temperature probe has existed since 1805 and has since gone through multiple iterations including the electronic clinical thermometer in 1954 [15, 16]. The oral probe is sensitive when placed correctly into the sublingual pouch next to the sublingual artery [17]. It can be altered by salivation, previous intake of hot or cold food, smoking, and rapid breathing [17]. Giuliano et al. [18] evaluated the Welch-Allyn oral thermometer (model 670; Welch Allyn, Inc., San Diego, CA, USA) in 72 ICU patients and found a significantly lower variability in temperature values compared with the tympanic probe. They concluded that oral monitoring should be considered a first-choice device if a PAC is not warranted.

The tympanic infrared probe should be considered next since it is easily assessed from two identical sites and optimally located 3.5 cm from the hypothalamus. However, the temperature of the tympanic membrane, and not the brain, can be influenced by convective air currents generated during resuscitation efforts [19]. Tympanic temperatures should only be considered for monitoring when oral temperature is not feasible.

A noninvasive, disposable, medical thermometer, based on zero-heat-flux thermometry technology (3 M, St. Paul, MN, USA) is now available in North America. The probe’s temperature is controlled to perfectly insulate the skin under the probe, which allows the warmer core tissue to warm the adjacent skin. In a study by Eshraghi et al. [20], the zero-heat-flux thermometry technology matched the PAC within 0.2 °C during cardiac surgery. This device has not been studied in trauma and requires further evaluation.

Cork et al. [21] evaluated the use of seven temperature monitors during anesthesia and compared their accuracy with tympanic measurements. Esophageal and nasopharyngeal temperatures were the most accurate while axillary temperatures were consistently 1.5–1.9 °C below tympanic temperatures. Precision of measurements using the nasopharynx, esophagus, and bladder was found to be superior to those obtained in the axilla, forehead, or rectum.

Rectal, bladder, and axillary measurement techniques are each less accurate than the methods already discussed [22]. Rectal values have been shown to lag behind core temperature in cardiopulmonary bypass (CPB). As such, using the rectal temperature is considered an “intermediate” measuring technique because it is not representative of the true core temperature [22, 23]. Bladder probes and axillary measurements are even less consistent, up to almost 1 °C in variability [24]. Each technique can be disturbed by external temperatures, local blood flow, and incorrect placement [25]. Many measurements are considered insensitive and therefore the most practical technique with the best precision should be applied.

### The physiological effects of hypothermia

#### Shivering and nonshivering thermogenesis

Nonshivering thermogenesis is important in infants because it increases metabolic heat without generating mechanical work. In adults, however, the hypothalamus stimulates skeletal muscles to shiver, which produces heat as a byproduct of cellular respiration [26]. In the absence of shivering (i.e., when paralyzed with neuromuscular blocking agents) the metabolic rate will decrease by 8 % for each degree of heat lost [26].

### Neurological effects

Cerebral metabolism in adults decreases by 7 % for each lost degree in heat. Patients will become confused, uncoordinated, and somnolent to eventually comatose at around 30 °C [27]. Below 27 °C, there is a loss of deep tendon reflexes and pupillary reflexes and eventually the neurological center becomes depressed. In this manner, a diagnosis of brain death cannot be made while a patient has severe hypothermia; patients must be rewarmed to 34 °C [28].

### Cardiovascular effects

Mild hypothermia (<36 °C) causes increased sympathetic tone, heart rate, blood pressure, and cardiac output while moderate hypothermia will depress cardiac activity [24, 27]. At 34 °C, hypothermia impairs diastolic relaxation. By 28 °C, bradycardia develops with a prolonged PR interval, Osborne waves, and T-wave inversions followed by ventricular fibrillation at 25 °C [27].

### Respiratory effects

Mild hypothermia can increase the respiratory rate, causing a decrease in the partial pressure of carbon dioxide (P_a_CO_2_). At moderate levels, airway reflexes are reduced, predisposing a patient to aspiration. At 32 °C, the medullary center becomes depressed, leading to a decrease in minute ventilation, increased secretions, and atelectasis [24, 29]. Gas exchange is not affected, but there is an increase in pulmonary vascular resistance and ventilation–perfusion mismatch.

### Renal effects

The initial increase in cardiac output, peripheral vascular resistance, and mean arterial pressure can lead to an in increase in renal blood flow and cold-induced diuresis [29]. With further heat loss the glomerular filtration rate decreases, reaching 50 % of normal at approximately 30 °C. Urine output does not decrease until 20 °C [29].

### Hematological effects

Clotting factor enzymes and platelets work optimally at 37 °C. Hypothermia impairs platelet function between 33 and 37 °C and the activity of clotting factors and fibrinogen synthesis below 33 °C [30]. Rohrer and Natale investigated the effects of temperature on coagulation. Partial thromboplastin time levels increased from 36.0 s at 37 °C to 39.4, 46.1, and 57.2 s at 34 °C, 31 °C, and 28 °C, respectively [31]. Temperatures below 33 °C also inhibit thrombin, glycoprotein Ib–IX complex, platelet aggregation, and thromboxane B2 production. One study investigated the reversibility of these effects in whole blood flow cytometric analysis and the complications were shown to resolve with rewarming back to 37 °C [32].

### Hypothermia and trauma-induced coagulopathy

Trauma-induced coagulopathy (TIC) is present in one-quarter of all severely injured patients and carries a 46 % mortality rate [33]. Several important processes, including the release of heparanoids from the endothelial glycocalyx, protein C activation, tissue plasminogen activator, hyperfibrinolysis, and platelet dysfunction, have been implicated [33]. The concept of early TIC is a new model that has not yet been entirely elucidated. However, several recent trials have identified a prolonged prothrombin time and therefore coagulopathy in the early time period after initial trauma in 25 % of patients [34]. There are seven mechanisms involved in its development: shock, tissue trauma, inflammation, acidemia, hemodilution, massive transfusion, and hypothermia [33]. Bukur et al. [35] retrospectively reviewed 21,023 trauma patients and found 44.6 % of prehospital patients had significant hypothermia, which can exacerbate all other mechanisms.

While shock is thought to be the primary driver of coagulopathy, tissue injury is still required for its initiation. Hypoperfusion causes tissues to become hypoxic and leads to lactic acidosis, which along with hypothermia decreases the activity of the coagulation cascade and increases fibrinolysis [36]. Sustained hypoperfusion increases thrombomodulin, which can increase thrombomodulin-bound thrombin that activates protein C, a systemic anticoagulant [36, 37].

Acidosis is defined as a pH less than 7.35. Engström et al. [38] used thromboelastography to show that the deficiency in clot formation progressively gets worse with increasing acidemia. TIC becomes more harmful, however, when acidemia is combined with hypothermia. There is a synergistic effect on the impairment of coagulation when acidosis is present with hypothermia but no significant change in clot formation with acidosis alone [39, 40]. Mitra et al. reviewed the outcome of major trauma patients with confirmed TIC. While they conceded that the existence of early coagulopathy does not establish futility, there were no survivors throughout an 8-year span in patients with extreme coagulopathy, hypothermia, and acidosis [41].

Early volume resuscitation is usually initiated with crystalloid solutions, which can worsen TIC further by dilution of clotting factors, dislodgement of clots, and inducing hypothermia [42, 43]. Even the assays completed at 37 °C underestimate coagulopathy when hypothermia is present [44]. Boyan [45] infused 3 liters of cold fluids into 25 trauma patients and found that the core temperature decreased and was associated with cardiac arrest in 12 patients. When the blood was warmed, however, the incidence of cardiac arrest in a matched group of patients was only 3 %.

### Risk factors for developing hypothermia and mortality

Risk factors should be classified according to their timing during resuscitation and are summarized in Table 3.

**Table 3:** Risk factors for hypothermia in trauma

Prehospital phase
Severity of injury
Head injury
Spinal cord injury
Shock
Extremes of age
Wet clothing
General anesthesia and prehospital intubation
Suspected medical conditions
Thyroid disease, adrenal disease, diabetes, cardiac dysfunction, hepatic disease, malnutrition, autonomic nervous system dysfunction
Hospital phase
Exposure
Cold intravenous fluids and blood products
Burns
General anesthesia
Epidural and spinal anesthesia
Observation phase
Size of surgery
General anesthesia >3 h
Intravenous crystalloids, blood products
SAPS II scores

*SAPS II* Simplified Acute Physiology II Score

### Prehospital phase

Lapostolle et al. prospectively determined factors associated with hypothermia in severely injured trauma patients in the prehospital setting. Risk factors included an elevated Revised Trauma Score (odds ratio (OR) 1.68), mobile unit temperature (OR 1.20), infusion fluid temperature (OR 1.17), and the presence of clothing (OR 0.40) [46].

The association between hypothermia and severity of injury is well described [46]. Blood loss, large open wounds, and significant head injury can disrupt temperature regulation, leaving the patient vulnerable to heat loss. Ireland et al. [47] reviewed 732 medical records of major trauma patients between January and December 2008, and discovered similar risk factors including prehospital intubation (OR 5.18), ISS (OR 1.04), and systolic blood pressure <100 mmHg (OR 3.04)—all markers of injury severity. Aitken et al. [48] also found the same risk factors; that is, ISS >40, a Glasgow Coma Score of 3, or ventilated and hypotensive on admission. Similarly, Arthurs et al. confirmed these findings at the 31st Combat Support Hospital of Fort Bliss, TX, USA. Although a causal relationship has not been identified, the presence of hypothermia in severe injury is probably both instrumental and ancillary in the physiologic deterioration of trauma injury [49].

Infusion fluid temperature has also been shown to be a risk factor for hypothermia. Farkash et al. prospectively studied the effect of prehospital fluid administration on temperature in combat patients. The principle risk factors included the use of cold fluids, open body cavities, and severity of injury (moderate injury 36.8 ± 1.0 °C, severe injury 35.8 ± 1.6 °C, *p* = 0.026) [50]. Lapostolle et al. found that the fluid infused was usually at a temperature below 21 °C [46]. In both studies, however, by using hemorrhage control and preventing unnecessary crystalloid administration, patients could be resuscitated with fluid volumes that do not result in hypothermia [46, 50].

Several patient characteristics are also considered risk factors. The most important is advanced patient age. Danzl et al. [9] described this association in a retrospective review where the rewarming rate was faster in patients who were younger than 59 years old (1.08 ± 1.39 °C/h vs. 0.75 ± 1.16 °C/h). This was attributed to their reduced cardiopulmonary reserve with impaired thermoregulation [51]. Other risk factors include spinal cord injury, drug use, homelessness, and psychiatric/medical conditions such as hypothyroidism, hypopituitarism, and hypoglycemia. However, these factors have never been studied in trauma [52].

### Hospital phase

Hospital risk factors are more commonly associated with resuscitation techniques including induction of anesthesia and prolonged surgery [53].

On arrival in the trauma bay, the trauma team is gowned and ready to manage life-threatening injuries. In many cases, patients are initially intubated on arrival in the emergency department because of hemodynamic instability, respiratory distress, or agitation. Core temperature typically decreases <1 °C within 30 min of induction of anesthesia because of redistribution [53]. Matsukawa et al. studied heat loss and discovered that the temperature decreased 1.6 ± 0.3 °C in the first hour of anesthesia and redistribution contributed 81 % to this decrease. Hypothermia was related to low environmental temperatures, infusion of cold fluids, ventilation with cold gases, absence of muscle movement, and subcutaneous vasodilation [53]. Langhelle et al. [54] retrospectively evaluated body temperature in 1292 trauma patients and found a significant difference in temperature between anesthetized and nonanesthetized patients (35.0 ± 2.1 °C vs. 36.2 ± 1.0 °C, *p* <0.001). Even mild hypothermia from anesthesia can increase blood loss and the risk for transfusion by 16 % and 22 %, respectively [55]. Anesthetized trauma patients should therefore be routinely managed with various warming methods to prevent anticipated heat loss [55].

During maintenance anesthesia, there is significant skin and organ exposure during surgery. Roe [56] first identified that the exposure and return of cold bowel to the peritoneal cavity causes significant heat loss. Poveda et al. revealed that a longer duration of surgery was inversely related to a decrease in core temperature (*r* = –0.43; *p* <0.05). As a result, surgeons have replaced prolonged abdominal exploration with simple packing to limit surgical bleeding and prevent heat loss [57]. However, the question of whether a routine warm peritoneal lavage would improve temperature control after surgical hemostasis has never been examined in detail.

### Observation phase

There is limited evidence assessing the prevention of hypothermia in the postresuscitation phase. Abelha et al. [58] determined the incidence of hypothermia on arrival in the ICU to be 57.8 %. Thus, hypothermia may be poorly recognized or inconsistently managed during previous phases of management. Warming devices, monitoring techniques, and higher early temperatures were considered protective. Independent risk factors for hypothermia among all postoperative ICU patients include the magnitude of the surgery, general anesthesia >3 h, cold fluids, and Simplified Acute Physiology II Score (SAPS II) [58]. These are the same risk factors as those described in the prehospital phase. Hypothermia, however, was not a risk factor for mortality in the ICU. This result supports the recommendation that attempts should be made to increase body temperature before surgery.

### Methodologies and evidence for the prevention of hypothermia

During the resuscitation process, hypothermia can be overlooked because more serious injuries require urgent intervention. Indeed, one of the core principles of trauma resuscitation is “E” for exposure to ensure complete identification of injuries and temperature levels. However, in many cases, warming devices are not available and temperature may not even be recorded [59]. In one major trauma center, the temperature was only documented in 38 % of all trauma admissions [59]. A structured approach to the prevention and management of hypothermia would improve efficient temperature regulation in the critically ill trauma patient.

Murad et al. [60] recently evaluated prehospital care and mortality by implementing a treatment protocol for 200 traffic casualties with severe injury (ISS >9) in Iraq. These patients received standardized treatment that included airway management, hemodynamic stabilization, and hypothermia prevention. Mortality decreased from 44 % to 8 % in the treatment cohort; however, it was later found that these patients had improved baseline physiological stability, which may have confounded the results [60].

Husum et al. [61] applied a prehospital hypothermia treatment protocol to 30 consecutive landmine casualties in Northern Iraq and Cambodia. This included early temperature documentation, warming blankets, warmed intravenous fluids, and prompt removal of wet clothing. The principal risk factor associated with hypothermia was the amount of warming received during the prehospital phase. The incidence of hypothermia decreased from 19 % to 3 % when this simple organized system was used (95 % confidence interval: –24.3 to –6.1 %) [61]. If left untreated, persistent hypothermia and TIC in the ICU is associated with higher mortality; therefore, early treatment may potentially improve outcomes after major trauma [62].

Emphasis should be on prevention first and treatment second because it becomes more difficult to rewarm the trauma patient once considerable heat is lost. Methods include passive external warming, active external rewarming, and active core or internal rewarming. A summary of the types of warming devices and a description of their use is presented in Table 4.

**Table 4:** Warming methods

Warming device	Manufacturer	Description	Heat transfer
Warming blanket	Bair Hugger 750, 505 (Arizant Healthcare Inc., Eden Prairie, MN, USA)	Air delivered to variety of blankets (upper, lower, full, torso, surgical access, pediatric, cardiac) at three settings: high (43 °C), medium (38 °C), low (32 °C)	Convection
	Equator (Smiths Medical ASD, Rockland, MA, USA)	Air delivered to adult and pediatric blankets. Settings: high (44 °C), medium (40 °C), low (36 °C)	Convection
	Thermacare TC3000 series (Gaymar Industries, Inc., Orchard Park, NY, USA)	Air delivered to adult and pediatric quilts: low (32 °C), medium (38 °C), high (43 °C), maximum (46 °C)	Convection
	WarmTouch 5200 (Nellcor, Pleasanton, CA, USA)	Air delivered to adult and pediatric blankets: high (42–46 °C), medium (36–40 °C), and low (30–34 °C)	Convection
Circulating water garment	Medi-Therm III (Gaymar Industries, Inc.)	Circulates water from the control unit to polymer hyper/hypothermia blankets. Manual settings: 4–42 °C; automatic: 30–39 °C	Conduction
	Blanketrol II Hyper–Hypothermia Water System (Cincinnati SubZero Products, Cincinnati, OH, USA)	Circulates water from the control unit to specialized blankets (adult and pediatric). Temperature range, 4–42 °C	Conduction
Heated air mattress	Polar Air (Augustine Medical, Inc., Eden Prairie, MN, USA)	Has not been shown to be effective [73] because only a limited amount of body surface area comes into contact with the mattress. Trauma patients may be vulnerable to burn injury	Conduction
Hot packs	Hot Cycle 1 (Sign Manufacturing Corporation, Fairfield, CA, USA)	Temperature at approximately 54.5 °C. Mean increase in temperature of 1.4 °C compared with a mean decrease of 0.3–0.6 °C in controls. Further research is necessary [64]	Conduction
Humidified gases	Heated Anesthesia Circuit (ANAPOD Westmed, Inc., Tucson, AZ, USA)	Delivery of warm, humidified gas can increase core temperature by 0.5–0.65 °C/h in injured patients [74]	Evaporation
Fluid warmer	General	Warmed fluids were found to increase temperature to 36.8 °C compared with 35.5 °C in nonwarmed patients [75]	Conduction
	Level 1 System H-1200, H-1000, H-1025, H-525, H-500, H-275, H-250 (Smiths Medical ASD, Rockland, MA, USA)	Aluminum heat exchanger with countercurrent 42 °C circulating water bath. Air detector/clamp	Conduction
	Hotline (Smiths Medical ASD)	Water bath heat exchange. Surrounds patient line with layer of 42 °C circulating fluid	Conduction
	FW600 Medi Temp III (Gaymar Industries, Inc.)	Dry heat exchange. Plastic disposable with aluminum heating plates (set point, 41 °C)	Conduction
	Thermal Angel TA-200 (Estill Medical Technologies, Dallas, TX, USA)	Battery-powered, portable in-line warmer. Outlet temperature, 38 ± 3 °C at flow rate 2–150 ml/min	Conduction
	Warmflo FW538 (Nellcor)	Dry heat exchange. Single-use metal cassette. Maximum flow rate, 500 ml/min	Conduction
Other	AV-300: CAVR – continuous	Rapid core rewarming. Circulates colder blood of patient through Level 1 heat exchanger and returns it to patient at Smiths Medical ASD	Conduction
	CairCooler (Pentatherm Ltd, Wakefield, UK)	Forced-air cooling system. Connects to forced-air blanket to deliver 10 °C air	Conduction
	Arctic Sun 2000 (Medivance, Louisville, CO, USA)	Circulating water temperature is controlled between 4 °C (39.2 °F) and 42 °C (107.6 °F) to achieve a preset target patient temperature	Conduction
	Lavage	The specific heat and rate of heat transfer in water is 32-fold greater than air, which permits effective hypothermia management [76]. The rate of rewarming is 1–3 °C per hour if done continuously	Conduction
	CPB	Hemodialysis (rate of rewarming is 2–3 °C/h), CPB using a heat exchanger (8–15 °C/h), and extracorporeal venovenous rewarming are other options for rewarming [77–79]. CPB is therefore the only technique that can also correct the hemodynamic stability of the patient and provides the greatest heat transfer	Conduction

*CAVR* continuous arteriovenous rewarming, *CPB* cardiopulmonary bypass

Adapted with permission from [80]

Passive rewarming allows the patient’s intrinsic heat-generating mechanisms to counteract heat loss. The natural rewarming rate is 1.20 °C/h while shivering can increase the rate up to 3.6 °C/h [63]. Passive mechanisms include removal of wet clothing, augmenting the environmental temperature, and applying a warm blanket. Watts et al., however, found that only active rewarming produced an increase in core temperature (+0.74 °C) in 134 trauma patients, while passive rewarming actually caused a decrease during transport. Therefore, usually passive mechanisms are more appropriate in mild hypothermia [64]. However, Lundgren et al. [65] investigated passive rewarming compared with active heat pads in a prospective trial in mild hypothermia. Mean temperatures insignificantly increased equally by 0.9 °C in each group.

Active external rewarming is used for patients with moderate hypothermia and no cardiac comorbidities. This includes the radiant warmer, electric blanket, forced warm air blanket, and heating pads [64, 66]. Kober et al. [67] evaluated 100 patients during prehospital transport and found that patients who received active resistive heating had less pain and anxiety and higher temperature levels (+0.8 °C vs. –0.4 °C). In severe hypothermia, however, peripheral vasoconstriction will limit the efficacy of external rewarming. One study investigated this theory by using a forced-air warmer in patients with temperatures below 30 °C. Fifteen patients were still effectively warmed to a temperature above 35 °C, demonstrating that external rewarming can still be considered in severe hypothermia [68].

Active internal rewarming is used for moderate to severe hypothermia. Internal rewarming restores temperature to normal levels at a faster rate than surface methods and is associated with more rapid normalization of cardiac output [69]. Active rewarming includes ventilation with humidified oxygen, warmed intravenous fluids, peritoneal lavage, and extracorporeal modalities such as dialysis, CPB, continuous arteriovenous rewarming (CAVR), and heparin-free extracorporeal life support [69]. Each method has its own inherent disadvantages and most have not been studied in trauma.

### A potential treatment algorithm

**Fig. 1:**
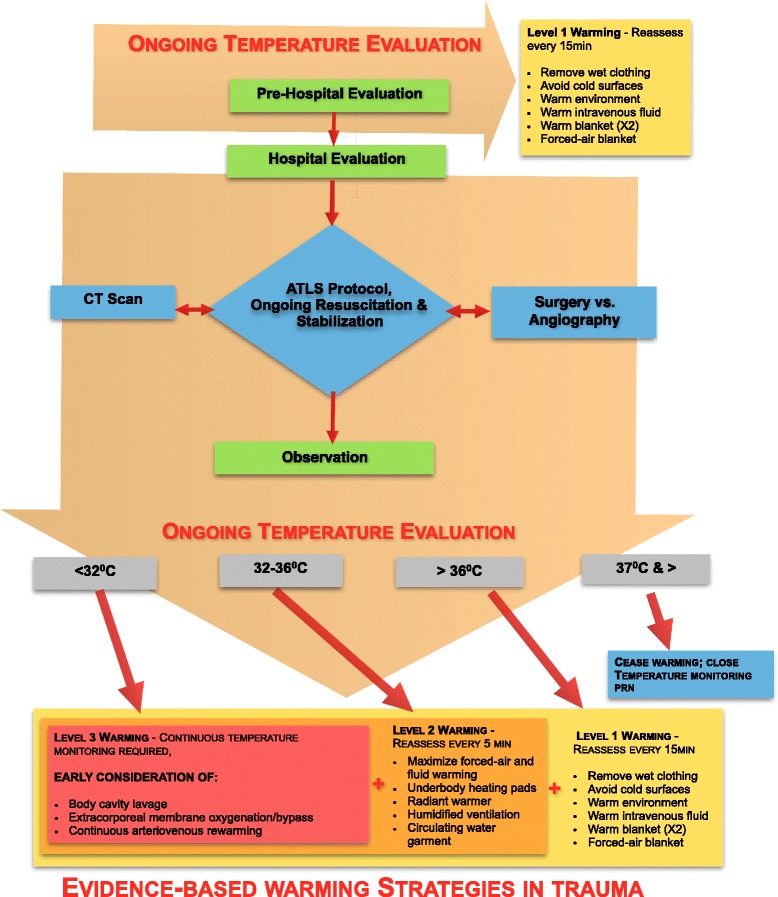
An algorithm for early goal-directed therapy for hypothermia in trauma. *ATLS* Advanced Trauma Life Support, *CT* computed tomography, *PRN* “pro re nata” (when necessary)

A potential strategy for early goal-directed therapy of hypothermia amongst trauma patients is detailed in Fig. 1. No randomized outcome data are available to provide a systematic review. Instead, we have described all available evidence and provided expert opinion to create a pragmatic treatment strategy that can be implemented in most major trauma centers. We intend to evaluate the efficacy of this protocol in an upcoming prospective trial based in Toronto, ON, Canada. This treatment protocol is divided into three sequential stages of resuscitation: prehospital, hospital, and observation phases.

Warming strategies have been grouped into graded “Levels” based on their evidence of successful application in trauma and ease of implementation. Level 1 includes both passive and active external strategies for mild hypothermia and can be easily applied. Level 2 includes heating pads, radiant heaters, warming blankets, and humidified gases. These techniques should be considered if a patient has a temperature between 32 and 36 °C. Level 3 rewarming is reserved for patients with severe hypothermia (<32 °C) whose condition is precarious enough to warrant invasive strategies including cavity lavage or extracorporeal circuits.

During the prehospital phase, first responders should be primarily focused on managing life-threatening injuries. In general, the patient’s core body temperature should be the guiding principle for the treatment modality. However, we recognize the difficulty in taking a measurement at this point and recommend all patients be immediately warmed using Level 1 techniques until arrival at the hospital. This includes the removal of wet clothing, warm blankets/forced-air warmer, and limiting cold fluid infusions. Furthermore, more invasive strategies, including extracorporeal circuits, require extensive training to operate and may not be available at the receiving hospital. In any case, emergency medical services should notify the receiving hospital to anticipate and plan for active warming strategies when indicated because Level 2 and Level 3 strategies involve significant set-up time.

In the trauma bay or hospital phase, a temperature should be recorded and rewarming resumed during the exposure stage of the primary survey. If the core temperature is above 36 °C, the patient should be covered with two warm blankets with regular temperature monitoring every 15 min as per Level 1 recommendations. If the temperature drops below 36 °C, the trauma team leader should initiate a warming strategy based on the patient’s recorded temperature. This includes adding Level 2 strategies with regular temperature monitoring every 5 min.

At this point, most trauma patients will then be transferred to the computed tomography room for diagnostic imaging, directly to the operating room, or to an observation unit—usually the ICU or the emergency room. There is considerable variation in the availability of different warming techniques, and therefore our algorithm should be customized to each patient’s injuries and hospital resources. Level 1 and Level 2 warming strategies should continue throughout the transfer process based on their feasibility and the availability of equipment. The techniques should be used in combination with each other if necessary and we support the option of changing to more invasive treatment levels if hypothermia is inadequately controlled.

After the patient has been transferred to an observation unit, warming protocols should continue based on the unit’s own protocols. If a unit does not have a warming protocol we suggest continuing with the same strategies as already outlined. Rewarming to a minimum core temperature of 36 °C is advisable prior to transfer unless the patient is stable and some semblance of temperature homeostasis is achieved. This will guarantee adequate warming before transferring the patient to another unit where rewarming in trauma may not be a priority. However, rewarming should cease after 37 °C because temperatures in this range are also associated with poor outcome and increased mortality [70].

## Conclusion

Hypothermia is considered an independent risk factor for mortality. This is related to its initial impairment of cardiovascular function, coagulation, and lactic acidosis followed by respiratory compromise. Risk factors in trauma include prolonged cavity exposure, cold fluids, environmental temperature, prehospital intubation, and severity of injury. Rewarming therapy should begin in the prehospital phase with passive and active warming strategies. This includes heating blankets, warm intravenous fluids, forced-air warmers, humidified gases, and CAVR. While these therapeutic modalities are usually readily available, consistent rewarming of trauma patients is still not effectively performed. We believe a systematic algorithmic approach to managing hypothermia in major trauma will encourage thorough rewarming of each patient and prevent its associated morbidity and mortality.

## Abbreviations

CAVR: continuous arteriovenous rewarming
CPB: cardiopulmonary bypass
ISS: Injury Severity Score
OR: odds ratio
PAC: pulmonary artery catheter
P_a_CO_2_: partial pressure of carbon dioxide
SAPS II: Simplified Acute Physiology II Score
TIC: trauma-induced coagulopathy
